# The Barriers to Cardiothoracic Surgery Training in Nigeria: Key Insights From Trainees

**DOI:** 10.7759/cureus.68923

**Published:** 2024-09-08

**Authors:** Chinedu Okoli, Obieze Nwanna-Nzewunwa, Arua Onyinyechukwu Adaeze, Ekene Etukokwu, Chinenye Okoli

**Affiliations:** 1 General Surgery, Institute for African Research, Innovation, and Education, Port Harcourt, NGA; 2 General Surgery, MaineHealth, Portland, USA; 3 Cardiothoracic Surgery, Institute for African Research, Innovation, and Education, Port Harcourt, NGA; 4 Cardiothoracic Surgery, UT Southwestern Medical Center, Dallas, USA; 5 Cardiothoracic Surgery, Federal University Teaching Hospital Owerri, Owerri, NGA; 6 Cardiothoracic Surgery, University of Nigeria Teaching Hospital, Enugu, NGA; 7 Family Medicine, Red Cross Society, Portland, USA

**Keywords:** barriers to training, cardiothoracic surgery, international collaboration and development, low case volume, nigeria, trainees

## Abstract

Aim

Cardiothoracic surgery has the potential to improve care in resource-deprived countries like Nigeria. This study analyzes the barriers to training in cardiothoracic surgery and gaps in the existing curriculum from a cardiothoracic surgery trainee perspective.

Methods

An online nationwide mixed-method cross-sectional survey was conducted. The participants were from a pool of senior residents in cardiothoracic surgery. A five-point Likert scale was utilized to rate and evaluate their training experiences. The motivations for choosing cardiothoracic surgery, gaps in the training curriculum, perceived barriers, and facilitators in their training programs were also assessed. The survey included closed and open-ended questions to capture quantitative data and qualitative insights. The quantitative data were analyzed using SPSS 21 (Armonk, NY: IBM Corp), while the qualitative data were analyzed using MAXQDA 24 (Berlin, Germany: VERBI Software).

Results

Sixteen senior cardiothoracic surgery residents completed the survey. Thematic grouping identified several key barriers, such as low case volume, lack of infrastructure and equipment, and reduced trainee autonomy during cases. The significant deficiencies in the existing curriculum are the absence of clear minimum competencies, lack of local and international collaboration, and robust cardiac training. In low-resource settings like Nigeria, government participation and improved funding, increased collaboration between local and international programs, and establishment of regional centers may offer solutions and successful implementation of cardiothoracic surgery training and improve access to surgical care.

Conclusion

Cardiothoracic trainees are highly interested in their training despite several barriers. Increased funding, collaboration, and infrastructure development will help improve the training experience.

## Introduction

Cardiothoracic surgery (CTS) is a highly specialized field requiring extensive training and hands-on experience. In many developing countries, including Nigeria, the training programs for CTS face significant challenges that impede the development of proficient surgeons [1-3]. These challenges include inadequate training infrastructure, limited access to basic surgical tools and technology, and insufficient funding for training programs and individual trainees [1-4].

The shortage of cardiothoracic (CT) surgeons in Nigeria is severe [5]. With a population of 200 million and a growing burden of cardiovascular diseases, there is an urgent need for well-trained CT surgeons to meet healthcare demands [6]. However, the number of accredited CT training institutions remains limited. Various challenges, such as the nature and quality of training and limited training opportunities, are believed to contribute to this inequity. These barriers affect the quality of training and lead to a brain drain, as many aspiring surgeons seek better opportunities abroad. Previous studies have highlighted these challenges, but they have not included the perspectives of trainees in Nigeria [1-4].

Despite the obvious critical need for CT surgeons, the current training landscape in Nigeria remains disparate and under-resourced. The disparity between the number of qualified trainers and trainees, outdated or unavailable surgical equipment, and many other factors severely hamper the practical experience necessary for proficiency in this demanding field [4]. Furthermore, there is a notable lack of simulation-based training, which is essential for honing technical and non-technical skills without compromising patient safety [7]. The current pathway to becoming a CT surgeon in Nigeria includes an initial minimum of 24-30 months of general surgery training followed by another minimum of 36-48 months of dedicated CTS training in an accredited institution after success in the membership examination of West African College of Surgeons and/or part 1 fellowship examination of National Postgraduate Medical College of Nigeria [8,9]. The residents in dedicated CTS training are referred to as senior registrars. The senior registrars, after meeting the requirements in the case logs and passing the fellowship examinations of either aforementioned college, qualify to become CT surgeons.

Addressing the previously mentioned gaps will require financial investment and strategic planning to ensure that training programs are well-structured, comprehensive, and aligned with global standards. Additionally, fostering a culture of mentorship and continuous professional development within existing medical institutions can provide trainees with the support and guidance they need to succeed. By implementing these measures, Nigeria can cultivate a new generation of skilled CT surgeons capable of addressing the country’s growing cardiovascular health challenges. Collaborative efforts between government bodies, healthcare institutions, and international partners can enhance the training environment. Nigeria can develop a robust CTS training program that meets international standards by improving infrastructure, providing adequate funding, and establishing standardized training protocols.

In order to improve CTS training, it is important to understand the challenges and areas for improvement from the perspective of the trainees. This study aimed to investigate the barriers to and facilitators of CTS training in Nigeria from the perspective of trainees and to propose effective solutions. The findings will be used to advocate for changes in health policy in Nigeria, addressing the observed challenges and creating policies to support the development of CT practice in the country.

## Materials and methods

Study design and data collection

This study employed a mixed-method cross-sectional survey design to assess the barriers and facilitators of CTS in Nigeria. The online survey was created to survey senior registrars in CTS across various training institutions in Nigeria. The survey was distributed twice via email in early 2024 and mid-2024 to all current CT surgical trainees. The survey URL was provided in the email sent to potential participants, described above. Individuals were not asked to identify themselves. Participants were informed that survey completion served as consent to participate in the study. Not all participants answered all questions.

Study survey development and data analysis

The survey questionnaire was specifically developed for this study. The survey questions were taken from previous articles on barriers to the career and practice of CTS in low- and middle-income countries [4-5,10]. These questions were then refined with the help of global health experts. Some questions on curriculum gaps, training barriers, and Likert scales were reworded to have a standard structure applicable to Nigerian CTS trainees. The survey was programmed and administered online to the initial pilot residents. After the initial pilot test, the questions were reviewed for wording and comprehensiveness, with suggested additional barriers from the experts included after review. The final survey included closed and open-ended questions to capture quantitative data and qualitative insights (questionnaire in the appendix). The data collected through the survey included the following: demographic information, reasons for choosing cardiothoracic surgery as a specialty, barriers to CTS training, gaps in the existing curriculum for CTS training, factors that could improve the cardiothoracic surgery training experience, and a Likert rating of trainees' satisfaction with the current CTS training. The quantitative data were tabulated using Microsoft Excel and analyzed using SPSS 21 (Armonk, NY: IBM Corp), while the qualitative data were analyzed using MAXQDA 24 (Berlin, Germany: VERBI Software). Data analysis included descriptive analyses.

Participants

This study's participants were senior registrars in CTS from accredited training institutions in Nigeria. Inclusion criteria included being a current senior registrar and having a minimum of one year of training experience. Exclusion criteria include non-completion of the survey and failure to give consent.

Ethical considerations

This study was reviewed and approved by the Institute for African Research, Innovation, and Education Institutional Review Board (#IRB-2024-IFARIE-0009). Before completing the survey, all participants provided informed consent. Participation was voluntary, and confidentiality was ensured by anonymizing the responses. The subjects were free to skip any question they wanted.

## Results

Of the 35 CT senior registrars in training in accredited centers in Nigeria, 16 completed the questionnaire (45.7% response rate). A total of 12.5% of the respondents declined to name their home institution, while the remaining came from nine tertiary hospitals, of which 57.1% were from institutions in Northern Nigeria. A total of 62.5% of the participants were in the 35-39 years age group, and 75.0% had been in training for three or more years. Only one female responded to the survey. Notably, 93.8% of the respondents were married. Further demographic data on the survey respondents are summarized in Table 1.

**Table 1:** Distribution of studied participants according to their age, years of training level, and marital status. CT: cardiothoracic

Social demographics		Frequency (%)
Age	30-34 years	1 (6.3)
	35-39 years	10 (62.5)
	40 years and above	5 (31.3)
Gender	Female	1 (6.3)
	Male	15 (93.8)
Marital status	Married	15 (93.8)
	Single	1 (6.3)
Years as a CT senior registrar	1 year	1 (6.3)
	2 years	3 (18.8)
	3 years	4 (25.0)
	4 years	1 (6.3)
	5 years	1 (6.3)
	>5 years	6 (37.5)

Reasons for choosing CTS

Passion and Interest

A total of 61.1% of the respondents reported passion and interest as the main reasons that attracted them to CTS. Their passion and interest are due to the uniqueness of CTS, their love for the chest and its contents, and their interest in surgery and cardiology. Meanwhile, 11.1% of the respondents mentioned the desire to help fill the manpower deficiency in the specialty and serve the subset of patients who often go without proper diagnosis and intervention as the reason for starting CTS training. Other indications are shown in Table 2.

**Table 2:** Reason to become a cardiothoracic surgeon. *Multiple response sets.

Reason to become a cardiothoracic surgeon	Frequency (%)*
Passion and interest	11 (61.1)
Desire to serve and fill the workforce gap	2 (11.1)
Sense of belonging	1 (5.6)
Prestige and honor	1 (5.6)
Mentorship	1 (5.6)
Specific interest	1 (5.6)
Charisma	1 (5.6)

Barriers to training in CTS

The participants identified several barriers to CTS training (Table 3). The top barriers were an inadequate number of cases (n=11; 34.4%), training infrastructure and resources (n=5; 15.6%), and reduced trainee autonomy during cases (n=3; 9.4%). Other reported barriers to CTS training are shown in Table 3. Low case volume was the most common barrier reported by the respondents. However, the reasons for the low volume of cases differ. The lack of health financing is the common theme reported as the cause of the inadequate case volume. For instance, one of the respondents stated, “Lack of insurance for patients and out-of-pocket expenditure by patients leading to delayed presentation and no presentation.” Another respondent stated that “Disharmony between the cardiologist and cardiac surgeons on optimal management results in poor referral and communication leading to delayed or no referral.” A resident reported that “few procedures are done due to unavailable instruments.

**Table 3:** Barriers to CTS training. *Multiple response sets. CT: cardiothoracic; CTS: cardiothoracic surgery

Barriers to CT surgery training	Frequency (%)*
Low volume of cases	11 (34.4)
Training infrastructure and resources	5 (15.6)
Reduced trainee autonomy during procedures	3 (9.4)
Funding and sponsorship	1 (3.1)
Lack of simulation lab	1 (3.1)
I prefer not to say	1 (3.1)
limited minimal invasive surgery exposure	1 (3.1)
Poor referral system	1 (3.1)
High cost of surgery	1 (3.1)
Poor curriculum	1 (3.1)
Government and administrative support	1 (3.1)
Lack of support system	1 (3.1)
Inadequate training of support staff	1 (3.1)
Lack of insurance	1 (3.1)
Inadequate training	1 (3.1)
Limited trainers experience	1 (3.1)

Gaps in the current curriculum

The most common deficiencies in the existing curriculum reported included a lack of clear minimum competence required to complete training (n=2, 12.5%), limited cardiac surgery rotations (n=2, 12.5%), and inadequate collaborations with local or international institutions (n=2, 12.5%). Other gaps reported in the existing curriculum are shown in Table 4.

**Table 4:** Gaps in the existing CTS training curriculum. *Multiple response sets. CT: cardiothoracic; CTS: cardiothoracic surgery

Gaps in the current CTS training curriculum	Frequency (%)*
Lack of clear minimum competence to become a CT surgeon	2 (12.5)
Limited cardiac surgery rotations	2 (12.5)
Lack of collaboration	2 (12.5)
Lack of integrated pathway	1 (6.3)
Current requirements for foreign rotation	1 (6.3)
Lack of research support	1 (6.3)
Low case volume	1 (6.3)
No gaps in the current curriculum	1 (6.3)
Poor trainer-trainee interactions	1 (6.3)
Inadequate pulmonology and intensive care unit rotation	1 (6.3)
Training not reflecting local needs	1 (6.3)
Lack of simulators for technical skills training	1 (6.3)
Lack of interval assessment	1 (6.3)

Factors that will improve CTS training experience

The top reported factors improving the CTS training experience include collaboration and networking (n=5, 18.5%) and sponsorship and funding of CTS residents (n=5, 18.5%). Respondents also reported that having fully accredited training programs (n=3, 11.1%) and fellowship exam centers in Nigeria (n=3, 11.1%) will also help to improve the training experience. Three (11.11%) respondents do not think the current training program needs improvement. Other reported factors that will improve the CTS training experience are shown in Table 5. Collaboration and networking with CT residents' sponsorships were the two factors that were suggested to improve the CTS experience. However, the respondents differ on how the collaboration and the sponsorship should be implemented. For example, one of the respondents stated that “Collaboration/affiliation of local institutions with foreign centers will facilitate subsidized additional training cost." Another resident thinks that government collaboration is more important than collaboration with foreign institutions, as shown by “government collaboration and public awareness of the current gap in accessing CT surgical expertise.” Another respondent stated that the sponsorship should be used to pay for society dues, as shown in “collaboration with other associations and discount membership for residents as well as a partnership with other international training.”

**Table 5:** Factors that will improve cardiothoracic surgery training experience. *Multiple response sets.

Factors that will improve cardiothoracic surgery training experience	Frequency (%)*
Collaboration and networking	5 (18.5)
Sponsorship of CT residents	5 (18.5)
Fully accredited programs and fellowship exam centers in Nigeria	3 (11.1)
Infrastructure development	3 (11.1)
Increased government participation	2 (7.4)
Regular audits to evaluate care	1 (3.7)
Minimal invasive surgery exposure	1 (3.7)
Simulation training	1 (3.7)
Improving trainee interest	1 (3.7)
Insurance	1 (3.7)
Structured resident training	1 (3.7)
Nothing to improve	3 (11.1)

Likert rating of trainee’s satisfaction with current training in CTS

None of the respondents was satisfied with the current training, with 75.0% reporting dissatisfaction. 81.2% of the respondents agreed that hands-on training is essential to their training, and 93.8% agreed that improved financing and sponsorship are essential. A total of 87.5% of the respondents agreed that having state-of-the-art surgical equipment will enhance training, while 12.5% did not think this would be beneficial. A total of 68.8% of the respondents believe that research opportunities are essential to their training and help them understand their practice. Other ratings of trainees’s satisfaction with the current training are shown in Tables 6, 7.

**Table 6:** Likert rating of trainee’s satisfaction with current training in CTS - academics and research. CTS: cardiothoracic surgery

Evaluation of ongoing cardiothoracic surgery training in Nigeria - academics and research	Rating	Frequency (%)
How satisfied are you with the current resources available for cardiothoracic surgery in Nigeria?	Very satisfied	-
	Satisfied	-
	Neutral	4 (25.0)
	Dissatisfied	9 (56.3)
	Very dissatisfied	3 (18.8)
To what extent does hands-on experience help in your learning and skill development?	Essential	13 (81.2)
	Very beneficial	3 (18.8)
	Beneficial	-
	Neutral	-
	Not beneficial	-
How effective do you think a structured curriculum is for training?	Highly effective	12 (75.0)
	Effective	4 (25.0)
	Neutral	-
	Ineffective	-
	Very ineffective	-
How essential are research opportunities in improving your understanding and practice?	Essential	11 (68.8)
	Very beneficial	1 (6.3)
	Beneficial	3 (18.8)
	Neutral	1 (6.3)
	Not beneficial	-
To what extent do diverse surgical cases contribute to your overall learning experience?	Essential	9 (56.3)
	Very beneficial	6 (37.5)
	Beneficial	1 (6.3)
	Neutral	-
	Not beneficial	-

**Table 7:** Likert rating of trainee’s satisfaction with current training in CTS - non-academics and research. CTS: cardiothoracic surgery

Evaluation of ongoing CTS training in Nigeria - non-academics and research	Rating	Frequency (%)
Do you feel adequately supported by the mentorship program or senior surgeons during your training?	Strongly agree	3 (18.8)
	Agree	7 (43.8)
	Neutral	1 (6.3)
	Disagree	3 (18.8)
	Strongly disagree	2 (12.5)
How do financial constraints impact your pursuit of advanced training in CT surgery?	Significant impact	16 (100)
How beneficial are state-of-the-art surgical equipment in enhancing your training?	Very beneficial	14 (87.5)
	Beneficial	-
	Neutral	2 (12.5)
	Not beneficial	-
	Not relevant	-
How essential are research opportunities in improving your understanding and practice?	Essential	11 (68.8)
	Very beneficial	1 (6.3)
	Beneficial	3 (18.8)
	Neutral	1 (6.3)
	Not beneficial	-
In your opinion, how important is financial support for pursuing advanced training?	Very important	15 (93.8)
	Important	-
	Neutral	1 (6.3)
	Less important	-
	Not important	-
How beneficial do you find networking and collaboration opportunities in your training?	Very beneficial	10 (62.5)
	Beneficial	4 (25.0)
	Neutral	2 (12.5)
	not beneficial	-
	Not relevant	-
How important are continuing medical education programs in keeping you updated?	Very important	10 (62.5)
	Important	-
	Neutral	4 (25.0)
	Less important	1 (6.3)
	Not important	1 (6.3)
How supportive is the institutional environment in your growth and development?	Strongly supportive	6 (37.5)
	Supportive	1 (6.3)
	Neutral	5 (31.3)
	Not supportive	3 (18.8)
	Very unsupportive	1 (6.3)

## Discussion

Given the rising population and prevalence of cardiovascular diseases, CT surgical training has become more crucial in low-income countries such as Nigeria. However, it faces numerous unprecedented global challenges driven by rapid innovations, the development of new minimally invasive techniques, health inequality, and the high cost of procedures in the context of out-of-pocket payment in a population with limited insurance coverage. Our objective in this study was to characterize the viewpoints of CT surgical trainees regarding their training experience concerning barriers and factors that will improve training. First, our study highlighted that the majority of current CTS senior registrars chose CTS because of passion and interest. This finding contradicts earlier studies that showed that having role models and mentors available to provide career insights can help in the decision-making process for choosing a specialty, which is a critical factor in choosing cardiothoracic surgery (CTS) [11]. Additionally, Gasparini et al. reported that early operative exposure and mentorship were crucial factors in promoting a career in CTS in their study, while Coyan et al. proposed that early interaction between students and CT faculty/trainees, as well as early exposure opportunities, may increase interest in CT surgery [12,13]. However, only one (5.6%) reported that mentors were responsible for the decision. Our survey indicated that another essential reason for selecting CTS is the desire to serve and fill the manpower gap. Nigeria’s population is estimated to be over 200 million, with only 0.5 open surgeries performed per million people [14]. In contrast, 1243 procedures were performed per million people in European countries, such as Germany [15].

Our study identified many barriers that impede CTS training. The most formidable barrier current trainees face is the low volume of cases. This finding also has been reported in other studies [3,16]. The low patient volume could be due to various factors, including high treatment costs, delayed presentation making surgical intervention not a viable option, and lack of insurance coverage. The primary method of funding healthcare in Nigeria is through out-of-pocket expenditure by patients and their families [17]. This can result in delays in early diagnosis and treatment, unlike what is seen in developed countries. Respondents also reported challenges with poor training infrastructure and resources. Due to the increasing complexity of CTS procedures, the current facilities and equipment are inadequate. Falase et al. assessed the hindrances to the growth of CTS in a tertiary hospital in Nigeria and found limited manpower development, infrastructure, high surgery costs, and funding mechanisms for surgery to be contributing factors [3].

The current curriculum for CTS training requires success in the part 1 fellowship exam of either the National Postgraduate Medical College of Nigeria or the West African College of Surgeons, followed by a successful fellowship exam. Unfortunately, there are no fully accredited centers in Nigeria for cardiothoracic surgery training. Additionally, the part 2 fellowship exam of the West African College of Surgeons can only be taken overseas. To address these challenges, it is essential to establish facilities with modern simulators and simulation laboratories to provide comprehensive hands-on training. Government support in providing necessary instruments and skilled staff in training institutions is crucial for filling this training gap [5].

Collaboration and networking were some of the factors that could improve the training experience. Cardiac surgeries in Nigeria mostly rely on foreign cardiac surgery missions organized by various international humanitarian organizations [18]. These have facilitated a few centers transitioning to autonomous programs [19]. Some local teams, independent of foreign cardiac missions, now offer open-heart surgery. This can be an effective strategy to expand academic institutional participation in global surgery and increase the volume of specialized surgeons. With the implementation of such bidirectional partnerships, residents could look to their home institution for help in organizing international surgical opportunities, thereby overcoming a primary barrier to training. More inter-center collaboration locally with a central coordinated training structure for residency will help to eliminate the effect of low case volumes and enhance exposure.

Sponsorship and funding are other significant factors that can improve the resident training experience. Our respondents identified financial constraints as one of the barriers that impact training, with five (18.5%) reporting that increased funding and sponsorship will help improve the training experience. This is not unique to CT surgical trainees and has been identified as the primary barrier in a cohort of various surgical specialties, suggesting that funding is a significant factor that can improve the training experience. Programs and departmental leadership should prioritize funding opportunities to ensure that they do not present as stressors to residents who wish to go into cardiothoracic surgery training.

Limitations

There are several limitations to this study. One of the primary limitations is sampling bias. Although the survey was completed online, those who responded could be those who felt dissatisfied with the ongoing training and indicated a willingness to participate in the survey. Another limitation includes the small sample size and the potential for response bias. Despite these limitations, the study attempts to understand the barriers and challenges faced by trainees with diverse backgrounds and levels of training.

Future areas of study include evaluating barriers and motivating factors for training programs in eliciting their motivating factors and perceived barriers to providing adequate training. Also, future research could benefit from a larger sample size and a longitudinal design to track changes in perceptions and experiences over time.

## Conclusions

This study shows that many CT trainees are highly interested in CTS and are passionate about it. However, there are barriers to training, such as the low volume of cases, and inadequate training facilities and resources. Improving these conditions requires increased collaboration, networking, government participation, sponsorship, and funding. By doing so, we can create new opportunities for trainees to enhance their training experience, ultimately improving access to much-needed CT surgical care in Nigeria.

## Appendices

Institute for African Research, Innovation, and Education: trainee’s perspective on cardiothoracic surgery training in Nigeria

A Qualitative Survey on Barriers and Facilitators

1. Name

2. Age range

3. Gender

a. Male

b. Female

c. Do not want to say

4. Marital status

a. Single

b. Divorced

c. Married

5. Name of your home training institution

6. For how many years have you been a cardiothoracic senior registrar?

a. 1 year

b. 2 years

c. 3 years

d. 4 years

e. 5 years

f. > 5years

7. Why did you choose cardiothoracic surgery?

8. How satisfied are you with Nigeria's current resources for cardiothoracic surgery training?

a. Very satisfied

b. Satisfied

c. Neutral

d. Dissatisfied

e. Very dissatisfied

9. What are your main challenges in accessing hands-on surgical training opportunities in cardiothoracic surgery?

10. Please specify any challenges you face

11. Do you feel adequately supported by mentorship programs or senior surgeons during your training?

a. Strongly agree

b. Agree

c. Neutral

d. Disagree

e. Strongly disagree

12. How do financial constraints impact your pursuit of advanced training in cardiothoracic surgery?

a. Significant impact

b. Moderate impact

c. Minor impact

d. No impact

e. Not applicable

13. Are there specific gaps in the curriculum that you believe should be addressed to improve training in Nigeria?

14. Do you face any cultural or societal barriers that hinder your progress in pursuing a career in cardiothoracic surgery?

a. Yes

b. No

15. How effective do you find networking opportunities available for cardiothoracic surgery residents?

a. Very effective

b. Effective

c. Neutral

d. Ineffective

e. Very ineffective

16. Are there any geographical or logistical challenges affecting your participation in related programs?

a. Yes

b. No

17. Have you encountered discrimination or bias during your training?

a. Yes

b. No

18. What improvements or initiatives do you believe would enhance training and address existing barriers?

19. Your suggestions for improvement

20. How important is access to experienced mentors for your training?

a. Very important

b. Important

c. Neutral

d. Less important

e. Not important

21. How beneficial do you find state-of-the-art surgical equipment in enhancing your training?

a. Very beneficial

b. Beneficial

c. Neutral

d. Not beneficial

e. Not relevant

22. To what extent does hands-on surgical experience contribute to your learning and skill development?

a. Essential

b. Very beneficial

c. Beneficial

d. Neutral

e. Not beneficial

23. How effective do you think a structured curriculum is for your training?

a. Highly effective

b. Effective

c. Neutral

d. Ineffective

e. Very ineffective

24. How essential are research opportunities in improving your understanding and practice?

a. Essential

b. Very beneficial

c. Beneficial

d. Neutral

e. Not beneficial

25. In your opinion, how important is financial support for pursuing advanced training?

a. Very important

b. Important

c. Neutral

d. Less important

e. Not important

26. How beneficial do you find networking and collaboration opportunities in your training?

a. Very beneficial

b. Beneficial

c. Neutral

d. Not beneficial

e. Not relevant

27. To what extent do diverse surgical cases contribute to your overall learning experience?

a. Essential

b. Very beneficial

c. Beneficial

d. Neutral

e. Not beneficial

28. How important are continuing medical education (CME) programs in keeping you updated?

a. Very important

b. Important

c. Neutral

d. Less important

e. Not important

29. How supportive is the institutional environment in fostering your growth and development?

a. Strongly supportive

b. Supportive

c. Neutral

d. Not supportive

e. Very unsupportive

30. Suggestions for improving cardiothoracic surgery training

31. Other comments
